# Supplementary material for: Learnt representations of proteins can be used for accurate prediction of small molecule binding sites on experimentally determined and predicted protein structures
Source: J Cheminform. 2024 Mar 14;16:32. doi: 10.1186/s13321-024-00821-4 (PMC10941399; doi:10.1186/s13321-024-00821-4)
Supplement: Supplementary file 1 — Additional file 1: Table 1. Top-1 success rate on training set using varying distance thresholds. Table 2. Success rate of binding site prediction of IF-SitePred and commonly used existingmethods. Table 3. Success rates and error estimations on the PDB structures of the HAP set. Table 4. Success rates and error estimations on the AF2 structures of the HAP set. Table 5. Success rates and error estimations on the PDB structures of the HAP-smallset. Table 6. Success rates and error estimations on the AF2 structures of the HAP-small set. Table 7. A comparison of ligand similarity to training set with success rates. Figure 1. Alphafold prediction confidence and prediction success. Figure 2. Global all-atom RMSD and ligand-binding site all-atom RMSD in MD structures. Figure 3. A comparison of the benefits of combining predictions for multiple structures. [file 13321_2024_821_MOESM1_ESM.pdf]

Additional File

Table 1: Top-1 success rate on training set using varying distance thresholds

min distance to protein	max distance to protein	distance for relabelling	clustering distance	success rate
2.5	5.5	4	1.6	0.99
2.5	5.5	4	1.7	0.99
2.5	5.5	4	1.8	0.99
2.5	5.5	4.5	1.6	0.99
2.5	5.5	4.5	1.7	0.99
2.5	5.5	4.5	1.8	0.99
2.5	5.5	5	1.6	0.99
2.5	5.5	5	1.7	0.99
2.5	5.5	5	1.8	0.99
2.5	6	4	1.6	0.99
2.5	6	4	1.7	0.99
2.5	6	4	1.8	0.99
2.5	6	4.5	1.6	0.99
2.5	6	4.5	1.7	0.99
2.5	6	4.5	1.8	0.99
2.5	6	5	1.6	0.99
2.5	6	5	1.7	0.99
2.5	6	5	1.8	0.99
2.5	6.5	4	1.6	0.99
2.5	6.5	4	1.7	0.99
2.5	6.5	4	1.8	0.99
2.5	6.5	4.5	1.6	0.99
2.5	6.5	4.5	1.7	0.99
2.5	6.5	4.5	1.8	0.99
2.5	6.5	5	1.6	0.99
2.5	6.5	5	1.7	0.99
2.5	6.5	5	1.8	0.99
3	5.5	4	1.6	0.97
3	5.5	4	1.7	0.97
3	5.5	4	1.8	0.97
3	5.5	4.5	1.6	0.99
3	5.5	4.5	1.7	0.99
3	5.5	4.5	1.8	0.99
3	5.5	5	1.6	1.0
3	5.5	5	1.7	1.0
3	5.5	5	1.8	1.0
3	6	4	1.6	0.97
3	6	4	1.7	0.97
3	6	4	1.8	0.97
3	6	4.5	1.6	0.99
3	6	4.5	1.7	0.99
3	6	4.5	1.8	0.99
3	6	5	1.6	1.0
3	6	5	1.7	1.0
3	6	5	1.8	1.0
3	6.5	4	1.6	0.97
3	6.5	4	1.7	0.97
3	6.5	4	1.8	0.97
3	6.5	4.5	1.6	0.99
3	6.5	4.5	1.7	0.99
3	6.5	4.5	1.8	0.99
3	6.5	5	1.6	1.0
3	6.5	5	1.7	1.0
3	6.5	5	1.8	1.0
3.5	5.5	4	1.6	0.93
3.5	5.5	4	1.7	0.93
3.5	5.5	4	1.8	0.93
3.5	5.5	4.5	1.6	0.99
3.5	5.5	4.5	1.7	0.99
3.5	5.5	4.5	1.8	0.99
3.5	5.5	5	1.6	0.99
3.5	5.5	5	1.7	0.99
3.5	5.5	5	1.8	0.99
3.5	6	4	1.6	0.93
3.5	6	4	1.7	0.93
3.5	6	4	1.8	0.93
3.5	6	4.5	1.6	0.99
3.5	6	4.5	1.7	0.99
3.5	6	4.5	1.8	0.99
3.5	6	5	1.6	0.99
3.5	6	5	1.7	0.99
3.5	6	5	1.8	0.99
3.5	6.5	4	1.6	0.93
3.5	6.5	4	1.7	0.93
3.5	6.5	4	1.8	0.93
3.5	6.5	4.5	1.6	0.99
3.5	6.5	4.5	1.7	0.99
3.5	6.5	4.5	1.8	0.99
3.5	6.5	5	1.6	0.99
3.5	6.5	5	1.7	0.99
3.5	6.5	5	1.8	0.99

Table 1: Success rate is largely stable to small perturbations in distance thresholds for point cloud generation and clustering. Thresholds for the following were varied: minimum distance from point to protein; maximum distance from point to protein; maximum distance from ligand-binding point to protein residue for residue to be relabelled as ligand-binding; maximum distance between two points within a cluster. Where the distance for relabelling residues as ligand-binding was lowered (4Å), success rate was reduced as low as 0.93, however all other combinations of thresholds yielded success rates of 0.99-1.00.

Table 2: Success rate of binding site prediction of IF-SitePred and commonly used existing methods

HAP: 688 proteins

PDB	baseline	IF-SitePred	FPocket	P2Rank	DeepPocket
Top 1	0.12	0.78	*0.75*	**0.81**	0.78
Top 2	0.17	**0.91**	*0.81*	0.90	0.87
Top 3	0.22	**0.93**	*0.83*	**0.93**	0.89

AF2	baseline	IF-SitePred	FPocket	P2Rank	DeepPocket
Top 1	0.09	0.77	*0.50*	**0.82**	0.78
Top 2	0.17	**0.89**	*0.60*	0.88	0.87
Top 3	0.20	**0.94**	*0.67*	0.89	0.90

HAP-small: 280 proteins

PDB	baseline	IF-SitePred	FPocket	P2Rank	DeepPocket
Top 1	0.12	0.76	*0.73*	**0.78**	0.75
Top 2	0.18	**0.90**	*0.80*	0.86	0.85
Top 3	0.24	**0.91**	*0.82*	**0.91**	0.88

AF2	baseline	IF-SitePred	FPocket	P2Rank	DeepPocket
Top 1	0.10	0.74	*0.48*	**0.76**	0.75
Top 2	0.17	**0.87**	*0.58*	0.85	0.86
Top 3	0.20	**0.94**	*0.65*	0.88	0.90

Table 2: IF-SitePred (trained with early stopping), P2Rank and DeepPocket are competitive across PDB and AF2 structures, whereas FPocket experiences a significant loss of performance on AF2 structures. Success rates of top-1, top-2 and top-3 binding site prediction as measured using DCA is shown, where success is defined as the centre of the predicted binding site being within 4Å of any ligand heavy atom. We show results for IF-SitePred, FPocket, P2Rank and DeepPocket on two test sets that contain PDB and AF2 structures respectively. For each test set, the highest success rate is shown in bold, and the lowest success rate is shown in italics.

Table 3: Success rates and error estimations on the PDB structures of the HAP set.

	Baseline	IF-SitePred		FPocket		P2Rank		DeepPocket	
	Success rate	Success rate	STD (95% CI)	Success rate	STD (95% CI)	Success rate	STD (95% CI)	Success rate	STD (95% CI)
Top 1	0.12	0.76	0.39-0.44	0.75	0.41-0.45	**0.81**	0.37-0.41	0.78	0.40-0.43
Top 2	0.17	0.89	0.25-0.32	0.81	0.37-0.42	**0.90**	0.28-0.34	0.87	0.31-0.36
Top 3	0.22	**0.93**	0.22-0.28	0.83	0.35-0.40	**0.93**	0.22-0.28	0.89	0.28-0.33

Table 3: Error estimations as calculated using bootstrapping of the predictions for PDB structures in the HAP set show that IF-SitePred and P2Rank have more consistent prediction success than FPocket and DeepPocket. The 95% confidence interval of the standard deviation for each success rate was calculated using SciPy's bootstrapping function. In general, error is estimated to be smaller where prediction accuracy is higher.

Table 4: Success rates and error estimations on the AF2 structures of the HAP set.

	Baseline	IF-SitePred		FPocket		P2Rank		DeepPocket	
	Success rate	Success rate	STD (95% CI)	Success rate	STD (95% CI)	Success rate	STD (95% CI)	Success rate	STD (95% CI)
Top 1	0.09	0.77	0.40-0.44	0.50	0.50-0.50	**0.81**	0.36-0.41	0.78	0.40-0.44
Top 2	0.17	**0.89**	0.28-0.34	0.60	0.48-0.50	0.88	0.29-0.36	0.87	0.31-0.37
Top 3	0.20	**0.94**	0.19-0.26	0.67	0.46-0.48	0.89	0.28-0.34	0.90	0.27-0.33

Table 4: Error estimations as calculated using bootstrapping of the predictions for AF2 structures in the HAP set show that IF-SitePred has more consistent prediction success than P2Rank, FPocket and DeepPocket. Confidence intervals were calculated as in Table 3.

Table 5: Success rates and error estimations on the PDB structures of the HAP-small set.

	Baseline	IF-SitePred		FPocket		P2Rank		DeepPocket	
	Success rate	Success rate	STD (95% CI)	Success rate	STD (95% CI)	Success rate	STD (95% CI)	Success rate	STD (95% CI)
Top 1	0.12	0.76	0.39-0.45	0.73	0.41-0.47	**0.78**	0.38-0.45	0.75	0.40-0.46
Top 2	0.18	**0.90**	0.26-0.35	0.80	0.36-0.43	0.86	0.30-0.39	0.85	0.32-0.40
Top 3	0.24	**0.91**	0.23-0.33	0.82	0.34-0.42	**0.91**	0.24-0.33	0.88	0.26-0.35

Table 5: Error estimations as calculated using bootstrapping of the predictions for PDB structures in the HAP-small set show that IF-SitePred and P2rank have more consistent prediction success than FPocket and DeepPocket. Confidence intervals were calculated as in Table 3.

Table 6: Success rates and error estimations on the AF2 structures of the HAP-small set.

	Baseline	IF-SitePred		FPocket		P2Rank		DeepPocket	
	Success rate	Success rate	STD (95% CI)	Success rate	STD (95% CI)	Success rate	STD (95% CI)	Success rate	STD (95% CI)
Top 1	0.10	0.74	0.41-0.46	0.48	0.50-0.50	**0.76**	0.39-0.46	0.75	0.40-0.46
Top 2	0.17	**0.87**	0.30-0.38	0.58	0.48-0.50	0.85	0.31-0.39	0.86	0.30-0.39
Top 3	0.20	**0.94**	0.19-0.30	0.65	0.46-0.49	0.88	0.28-0.37	0.90	0.26-0.35

Table 6: Error estimations as calculated using bootstrapping of the predictions for AF2 structures in the HAP-small set show that IF-SitePred has more consistent prediction success than P2Rank, FPocket and DeepPocket. Confidence intervals were calculated as in Table 3.

Table 7: A comparison of ligand similarity to training set with success rates

Ligand similarity to training set	IFSitePred	FPocket	P2Rank	DeepPocket
0.2-0.4	+0.05	-0.02	**-0.13**	**-0.21**
0.4-0.6	+0.03	+0.02	-0.02	0.00
0.6-0.8	*+0.35*	*+0.30*	**-0.10**	*+0.10*
0.8-1.0	-0.03	-0.04	+0.02	-0.03

Table 7: DeepPocket and P2Rank perform worse than average on sites that bind ligands significantly different to the training set. We calculated the Tanimoto similarity of the Morgan fingerprint (radius 2) for the most similar ligand in the training set to that binding each protein in the HAP-small set, and calculated the fraction difference between overall top-1 success rate (from Table **??**) for each 0.2 interval of ECFP similarity. Success rates differing by over 10% from the mean value are shown in bold (performance loss) or italic (performance gain).

Figure 1: Alphafold prediction confidence and prediction success

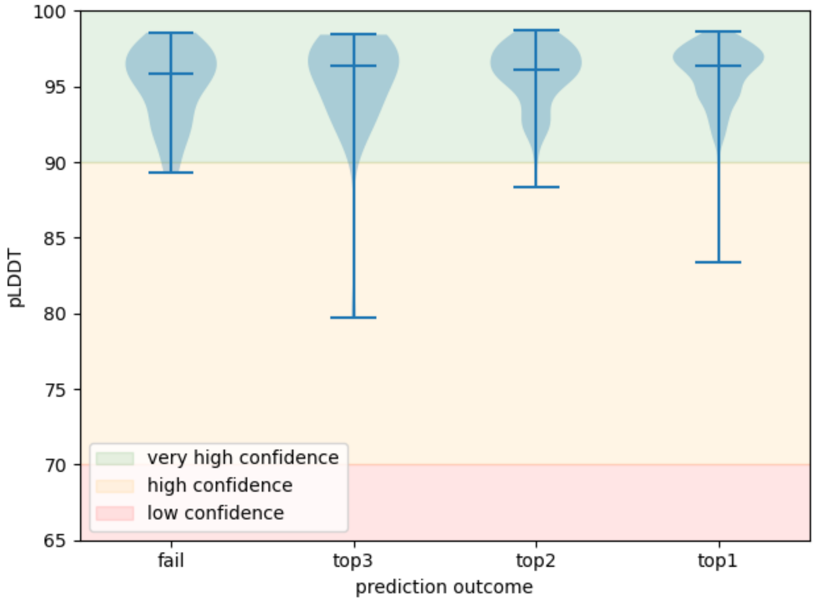

Figure 1: AlphaFold prediction confidence (pLDDT) does not vary between targets with top 1, top 2, top 3 success or failure in binding site prediction by IF-SitePred. While there are some outlying values, mean pLDDT is similar between levels of prediction success. The prediction confidence is very high compared with what would be expected for novel protein predictions.

Figure 2: Global all-atom RMSD and ligand-binding site all-atom RMSD in MD structures

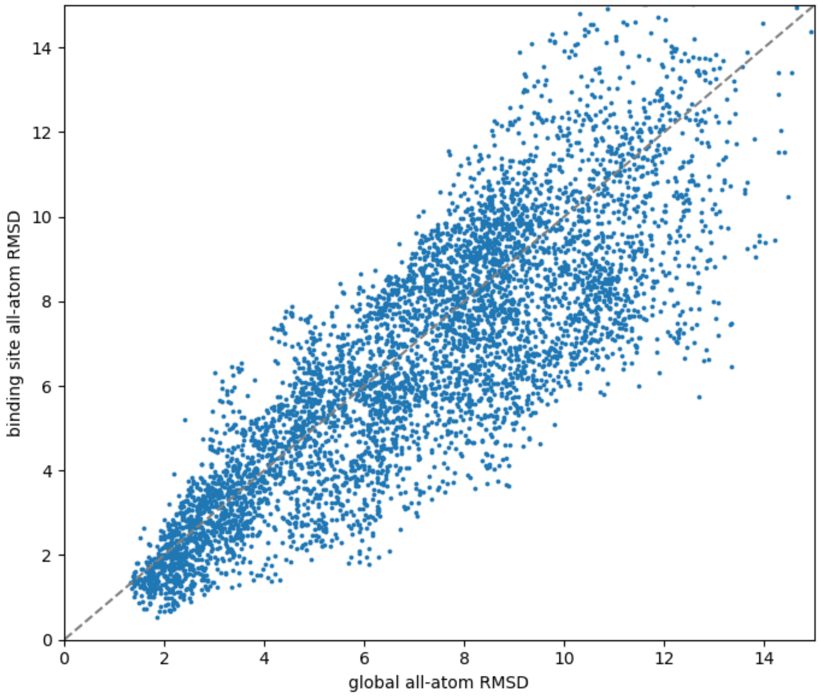

Figure 2: By comparing global all-atom RMSD in MD structures to all-atom RMSD for only atoms in the ligand-binding sites, we found that generally, local changes to these sites are of a similar level as the overall global changes experienced across the structure. Ligand-binding atoms were defined as those from residues with at least one heavy atom within 4Å of any ligand heavy atom. Global all-atom RMSD values were calculated using global alignment within PyMOL API with no rejection cycles. Ligand-binding site RMSD was calculated as the RMSD between relevant atoms given the global alignment.

Figure 3: A comparison of the benefits of combining predictions for multiple structures

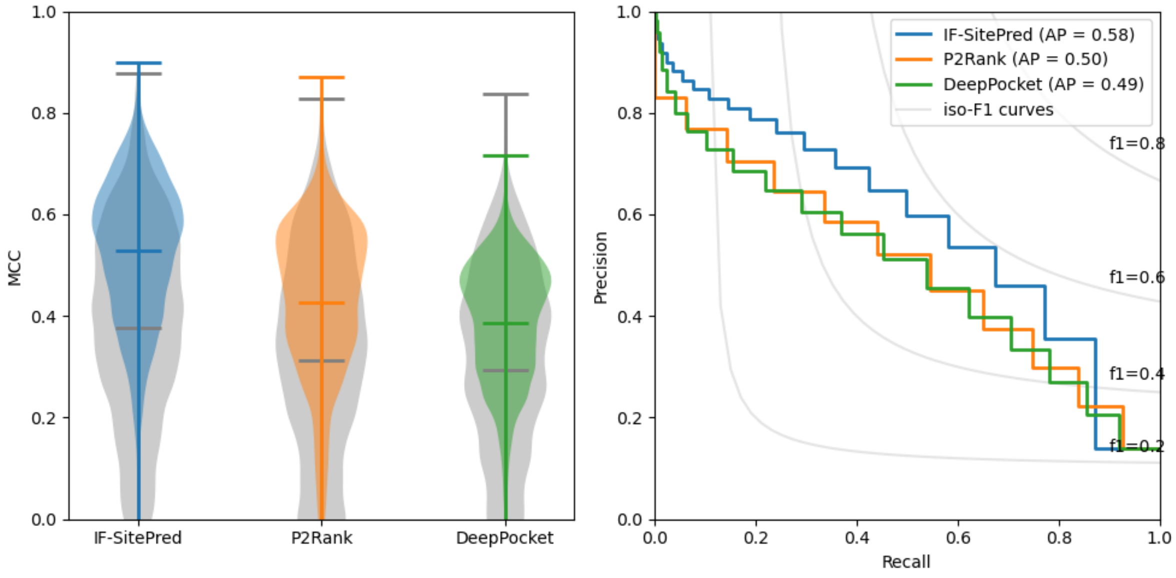

Figure 3: **Left:** Predictive power for binding residue annotation improves when predictions for 10 structures were combined for all three methods (blue, orange, green) compared to when predictions for single structures were used (grey). IF-SitePred had a slightly higher original MCC, and also saw the greatest improvement of the three methods. **Right:** Precision-recall curves for all three methods reveal that IF-SitePred has a higher average precision (AP) (0.58) than P2Rank (0.50) and DeepPocket (0.49). Iso-F1 curves are shown in grey, demonstrating that IF-SitePred achieves higher F1 scores across all probability thresholds.
